# Estrogen promotes the angiogenesis and osteogenesis of bone marrow stromal cells via regulating ESR1/RUNX2 axis

**DOI:** 10.29219/fnr.v70.13421

**Published:** 2026-01-12

**Authors:** Zhen Han, Chengjian Wei

**Affiliations:** Department of Orthopedics and Traumatology, Jiangsu Province Hospital of Chinese Medicine, The Affiliated Hospital of Nanjing University of Chinese Medicine, Nanjing, China

**Keywords:** osteoporosis, estrogen, ESR1, angiogenesis, osteogenesis

## Abstract

Osteoporosis (OP) is a common bone disease characterized by decreased bone mass and microarchitectural deterioration. This study aimed to investigate the effects of estrogen on OP. Bilateral ovariectomy (OVX) surgery was performed to establish the OP mouse model. Histological analysis was performed using hematoxylin and eosin (HE) staining and alizarin red S (ARS) staining. Angiogenetic factors were determined using enzyme-linked immunosorbent assay (ELISA). Messenger RNA (mRNA) levels were determined using quantitative reverse transcriptase-polymerase chain reaction (qRT-PCR). Protein expression was determined using Western blot. Cellular functions were analyzed using Transwell, tube formation, alkaline phosphatase staining, and ARS staining assay. The co-localization of estrogen receptor alpha (ESR1) and runt-related transcription factor 2 (RUNX2) was determined using the fluorescence in situ hybridization (FISH) assay. The interaction between ESR1 and RUNX2 was determined using the co-immunoprecipitation (Co-IP) assay. We found that 17β-estradiol (E2) alleviated the decrease in bone intensity and mass induced by OVX. E2 promoted the angiogenesis and osteogenesis of bone marrow stromal cells (BMSCs). Mechanistically, E2 predominantly upregulated ESR1. Moreover, mediated nuclear-localization of ESR1 promoted the interaction between ESR1 and RUNX2. Furthermore, ESR1 overexpression promoted the angiogenesis and osteogenesis of BMSCs. Estrogen exerts a protective effect on OP. Estrogen mediates the angiogenesis and osteogenesis of BMSCs by regulating the ESR1/RUNX2 axis.

## Popular scientific summary

1. Osteoporosis is characterized by bone loss and microarchitectural deterioration.
2. Increasing studies have demonstrated that estrogen deficiency is a key cause of osteoporosis.
3. ESR1 exerts protective function in osteoporosis.

Osteoporosis (OP) is a common bone disease characterized by decreased bone mass and microarchitectural deterioration (1). The decline in circulating estrogen levels is the primary driver of this accelerated bone loss (2, 3). While the canonical understanding of estrogen deficiency focuses on the upregulation of osteoclastic bone resorption (4, 5), a growing body of evidence underscores its equally critical role in suppressing bone formation and disrupting the bone’s vascular network (6–8).

Bone remodeling is a tightly coupled process reliant on the spatial and temporal coordination between osteoblasts (bone formation), osteoclasts (bone resorption), and the vasculature (9–11). New bone formation (osteogenesis) is intrinsically dependent on angiogenesis, the process of new blood vessel formation, which supplies oxygen, nutrients, hormones, and mesenchymal progenitors to the remodeling site (12, 13). Estrogen is a potent regulator of this ‘angiogenic-osteogenic coupling’ (14). Its deficiency leads to impaired angiogenesis, resulting in a hypovascular bone marrow environment that cannot support robust bone formation, thus contributing to the net bone loss observed in OP (15).

Bone marrow stromal cells (BMSCs), the multipotent progenitors for osteoblasts, reside in a perivascular niche and are master regulators of bone homeostasis (16, 17). Beyond their fate commitment to the osteoblastic lineage, BMSCs are a key source of paracrine angiogenic signals, most notably Vascular Endothelial Growth Factor (VEGF) (18, 19). The biological effects of estrogen are predominantly mediated through its nuclear receptors, Estrogen Receptor Alpha (ESR1) and Beta (ESR2) (20). ESR1 is the dominant subtype expressed in bone and is expressed in BMSCs, osteoblasts, osteocytes, and endothelial cells (21). ESR1 is known to promote the osteogenic differentiation of BMSCs. Crucially, emerging researches suggest that ESR1 activation stimulates the differentiation of BMSCs (22). This positions ESR1 in BMSCs as a potential master switch that synchronizes the bone formation program with the necessary expansion of the capillary network (23). However, a direct causal link demonstrating that the loss of ESR1 signaling specifically within the BMSC population is responsible for the uncoupling of angiogenesis and osteogenesis in estrogen-deficient OP remains to be fully elucidated.

This study aims to investigate the necessity of ESR1 in BMSCs for mediating estrogen’s protective effects on bone by genetically disrupting ESR1 in the mesenchymal lineage in a novel mouse model. We will characterize the resulting skeletal and vascular phenotype and employ *in vitro* assays to dissect the mechanisms governing ESR1-mediated crosstalk between osteogenesis and angiogenesis. This research will provide foundational insights into a novel mechanism of OP pathogenesis, identifying BMSC-ESR1 as a critical node for therapeutic intervention aimed at rejuvenating both bone and its blood supply.

## Materials and methods

### Experimental animals and estrogen-deficient model

Twelve-week-old female C57BL/6J mice (Charles River Laboratories) were maintained under specific-pathogen-free (SPF) conditions (12 h light/dark, 22 ± 1°C, 45% humidity). After 1-week acclimatization, mice were randomly assigned to: 1) Sham group (SHAM, *n* = 6), 2) Bilateral ovariectomy (OVX, *n* = 6), 3) OVX + 17β-estradiol replacement (OVX + E2, *n* = 6). OVX was performed via a dorsal approach under isoflurane anesthesia (3% induction, 1.5% maintenance). Buprenorphine (0.1 mg·kg^–1^ s.c.) was administered pre-operatively and every 12 h for 48 h. E2 replacement (20 µg·kg^–1^·day^–1^ s.c.) began 3 days after OVX and continued until sacrifice. Body weight was recorded weekly; vaginal cytology was monitored to confirm oestrous cycle arrest in OVX groups. Behavior tests were analyzed using three-point bending test of tibia bone midshafts. Mice were sacrificed 5 weeks post-surgery. All animal experiments were approved by the Ethical Committee of our hospital, and strictly implemented in compliance with the NIH Guide for the Care and Use of Laboratory Animals. All procedures were performed in accordance with ARRIVE guidelines.

### Histological analysis

Undecalcified femora were embedded in methyl-methacrylate and sectioned (5 µm) for Goldner’s Trichrome. Paraffin sections were stained for H&E and alizarin red S (ARS) staining. Quantification was performed in a blinded fashion using ImageJ.

### Micro-CT assay

Rats were scanned using SkyScan 1176 (Bruker, Germany). The levels of BV/TV, Tb.n, and Tb.Th were analyzed.

### Isolation and culture of BMSCs

Femora and tibiae were harvested under sterile conditions. Bone marrow was cultured with α-medium (MEM) containing 2 mM L-glutamine and 10% fetal bovine serum (FBS) (Gibco, USA). Cells were plated at 2 × 10^6^ nucleated cells·cm^–2^ and incubated at 37°C, 5% CO_2_. Non-adherent cells were moved out; medium was changed every 3 days. At 80% confluence (passage 0, P0), cells were trypsinized (0.25% Trypsin-ethylenediamine tetraacetic acid [EDTA]) and re-plated at 5 × 10^3^ cells·cm^–2^. All experiments used P3–P5 BMSCs. Then BMSCs were cultured with 10^–4^ μmol/L 17β-estradiol (Sigma-Aldrich, USA).

### Transfection

ESR1 overexpression plasmids and the empty vector were provided by GenePharma (Shanghai, China). ESR1 overexpression plasmids and the empty vector were transfected into BMSCs using Lipofectamine 2000 (Thermo Fisher Scientific, USA) for 48 h.

### ELISA

Mouse serum was obtained by collecting venous blood. The serum levels of procollagen type 1 N-terminal propeptide (P1NP) were quantified using an enzyme-linked immunosorbent assay (ELISA) kit (R&D Systems, USA).

### Quantitative reverse transcriptase-polymerase chain reaction (RT-PCR) for osteogenic and angiogenic genes

Total RNA was extracted using TRIzol and reverse-transcribed with SuperScript IV. Gene expression was quantified on a QuantStudio 6 Pro using SYBR Green. Primer sequences (Table S1) were validated for efficiency (90–110%) and single-product melt curves. Data were normalized to GAPDH and analyzed by 2^–ΔΔCt^.

### Western blotting

Proteins were extracted in radioimmunoprecipitation assay (RIPA) buffer + protease/phosphatase inhibitors. Equal amounts (20 µg) were separated on 10% sodium dodecyl sulfate-polyacrylamide gel electrophoresis (SDS-PAGE), transferred to polyvinylidene fluoride (PVDF), and probed with antibodies against runt-related transcription factor 2 (RUNX2) (ab2366391: 1,000, Abcam, UK), osteopontin (OPN) (ab214050; 1: 1,000, Abcam, UK), osteocalcin (OCN) (ab133612; 1: 1,000, Abcam, UK), vascular endothelial growth factor A (VEGFA) (ab46154; 1: 1,000, Abcam, UK), angiopoietin-1 (ANGPT1) (ab183701; 1: 10,000, Abcam, UK), and β-actin (ab213262; 1: 1,000, Abcam, UK). Later, the membranes were incubated with HRP-conjugated secondary antibodies (ab205718; 1: 10,000, Abcam, UK). The bands were visualized using ECL regents (Abcam, UK).

### FISH assay

Alexa Fluor 555-labeled RUNX2 ESR1 probes and Alexa Fluor 488-labeled ESR1 probes were designed and synthesized by RiboBio (Guangzhou, China). The probe signals were determined using fluorescence in situ hybridization (FISH) Kits (F32948 and F32947; Thermo Fisher Scientific, USA). Images were pictured by a fluorescence microscope (Leica, Germany).

### Co-IP assay

Cells were washed twice with ice-cold PBS and lysed in 1 mL modified RIPA buffer. Lysates were incubated and centrifuged. Supernatant protein concentration was determined by bicinchoninic acid assay (BCA) (Pierce, USA). Lysate was incubated with Protein A/G PLUS-Agarose beads (Santa Cruz, USA) conjugated with primary antibody (anti-ESR1, anti-RUNX2, or control immunoglobulin G [IgG]) overnight at 4°C. Beads were pelleted at 2,500 × g for 2 min and washed five times with 1 mL ice-cold lysis buffer. For stringent washing, the final two washes were performed with high-salt buffer (500 mM NaCl) to remove weak interactions. Immune complexes were eluted by boiling in 2× Laemmli sample buffer for 5 min at 95°C. Eluates were centrifuged. The immunoprecipitates (500 µL), along with inputs and other lysates, were collected and subjected to SDS-PAGE.

### Tube-formation assay

Passage 3 BMSCs were seeded (1 × 10^4^ cells/well) on growth-factor-reduced Matrigel (Corning) in endothelial basal medium (EBM-2) containing 2% FBS. Tube length, branch points, and mesh area were quantified at 6 h using ImageJ Angiogenesis Analyzer.

### Transwell assay

BMSCs were seeded in the upper chamber supplemented with α-MEM without PBS. To the lower chamber α-MEM + 2% FBS was added. After 24 h, the cells that remained in the upper chamber were wiped out. The migrated cells were pictured using an inverted microscope and quantified using ImageJ Angiogenesis Analyzer.

### Alkaline phosphatase staining

Alkaline phosphatase (ALP) staining was conducted using an Alkaline Phosphatase Color Development Kit (P0321S; Beyotime, China). Briefly, 500 µL of substrate solution was added per well and incubated for 30 min. The reaction was stopped by washing with distilled water. Images were captured using a DMi8 microscope (Leica, Germany)

### ARS staining

Cells were cultured in OM for 14 or 21 days. Then cells were fixed with 4% PFA and rinsed with distilled water. ARS solution (2%, pH 4.2) was prepared by dissolving ARS powder in distilled water, adjusting pH with 10% ammonium hydroxide, and filtering through 0.22 µm filter. One milliliter of stain was added. Unbound dye was removed. Plates were air-dried and imaged under bright-field microscopy. To quantify mineral deposition, 800 µL of 10% (w/v) cetylpyridinium chloride (CPC) in 10 mM sodium phosphate buffer (pH 7.0) was added and incubated for 1 h with gentle shaking. The supernatant (200 µL) was transferred to a 96-well plate and analyzed using a Varioskan™ LUX microplate reader (VL0L00D0; Thermo Fisher Scientific, USA) at the absorbance of 562 nm.

### Statistical analysis

Data were analyzed by GraphPad Prism 9.5, and presented as mean ± SD. Comparisons among multiple groups were analyzed by one-way ANOVA. Comparisons between two groups were analyzed by Student’s *t*-test. *P* < 0.05 was considered significant.

## Results

### Estrogen alleviates OP in vivo

**Fig. 1:**
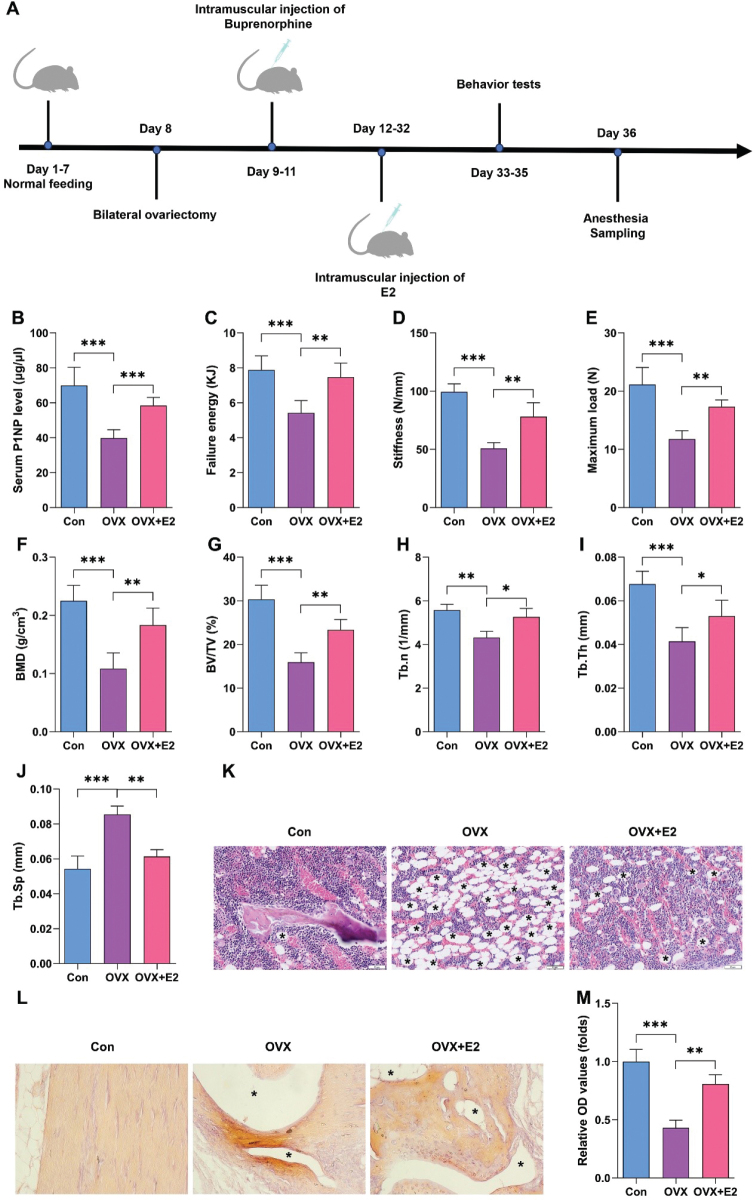
Estrogen alleviates osteoporosis (OP) *in vivo*. (A) The schematic diagram of the animal experiment. (B) Serum P1NP levels from tibia bone midshafts of 12-week-old female C57BL/6J mice were analyzed by ELISA assay. Fracture energy (C), stiffness (D), and maximal load (E) were analyzed using a three-point bending test of tibia bone midshafts from 12-week-old female C57BL/6J mice. Histomorphometric analysis was performed to evaluate BMD (F), BV/TV (G), Tb.n (H), Tb.Th (I), and Tb.Sp (J) in 12-week-old female C57BL/6J mice using micro-CT. Histological analysis was performed using HE (K) and ARS staining (L–M) in 12-week-old female C57BL/6J mice. *P < 0.05, **P < 0.01, ***P < 0.001.

To confirm the effects of estrogen on OP, mice were administrated with estrogen after OVX surgery. Figure 1A showed the schematic diagram of the animal experiment. We found that OVX surgery significantly reduced the serum levels of the bone formation marker P1NP (Fig. 1B), which was reversed by E2. E2 reversed OVX surgery-induced increase of energy to failure of the tibiae, stiffness, and maximum load (Fig. 1C–F). Micro-computed tomography (CT) analysis showed that estrogen administration significantly reversed the effects of OVX surgery and increased bone volume (BV)/total volume (TV), Tb.n, and Tb.Th, and also reduced Tb.Sp (Fig. 1G–J). Furthermore, histological analysis showed that estrogen administration significantly increased subchondral trabecular bone volume, alleviating OVX-induced loss due to thinner and fewer trabecular bones (Fig. 1K–M).

### Estrogen promotes angiogenesis of BMSCs

**Fig. 2:**
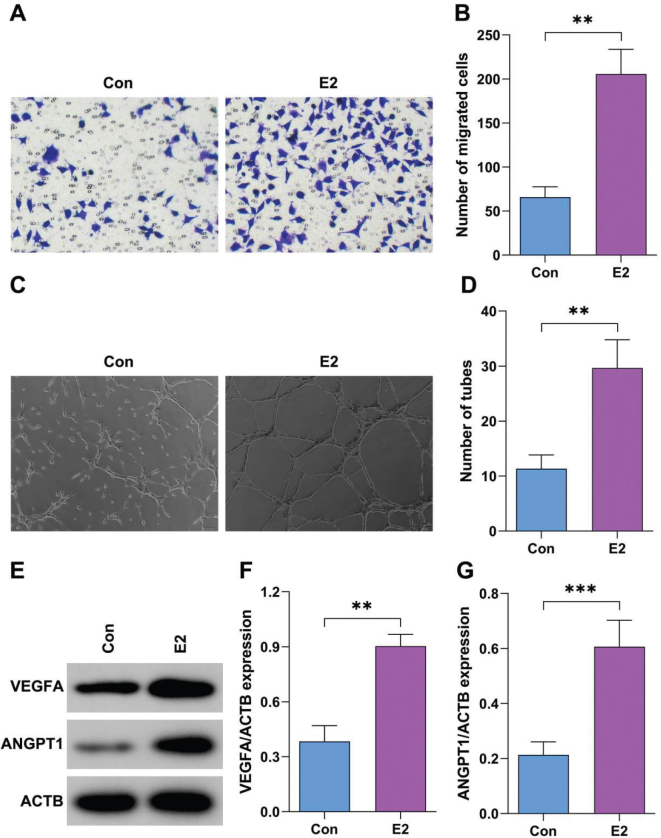
Estrogen promotes angiogenesis of bone marrow stromal cells (BMSCs). (A–B) The migration of BMSCs after treatment with E2 was analyzed by Transwell assay. (C–D) Tube formation of BMSCs after treatment with E2 was analyzed using tube formation assay. (E–G) Protein expression after treatment with E2 was analyzed using Western blot. **P < 0.01, ***P < 0.001.

To verify the effects of estrogen on OP, BMSCs were treated with estrogen. As shown in Fig. 2A–B, estrogen treatment significantly increased the migrated cells of BMSCs. Moreover, estrogen treatment significantly increased the branches of BMSCs (Fig. 2C–D). Furthermore, estrogen treatment significantly increased the protein expression of VEGFA and ANGPT1 (Fig. 2E–G).

### Estrogen promotes osteogenesis of BMSCs

**Fig. 3:**
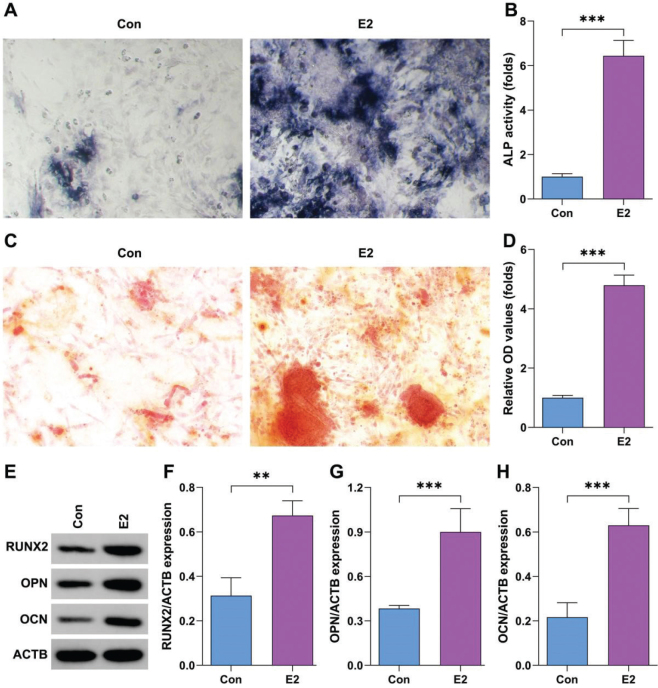
Estrogen promotes osteogenesis of bone marrow stromal cells (BMSCs). (A–B) The osteogenic ability of BMSCs after treatment with E2 was analyzed by alkaline phosphatase (ALP) staining. (C–D) Cellular mineralization of BMSCs after treatment with E2 was analyzed using alizarin red S (ARS) staining. (E–H) Protein expression in BMSCs after treatment with E2 was analyzed using Western blot. **P < 0.01, ***P < 0.001.

Estrogen treatment significantly promoted the osteogenic differentiation of BMSCs compared with control group (Fig. 3A–B). Moreover, estrogen treatment significantly mediated cellular mineralization of BMSCs compared with control group (Fig. 3C–D). Additionally, estrogen treatment significantly increased the protein expression of RUNX2, OPN, OCN (Fig. 3E–H).

### Estrogen predominantly upregulates ESR1 in OVX mouse model

**Fig. 4:**
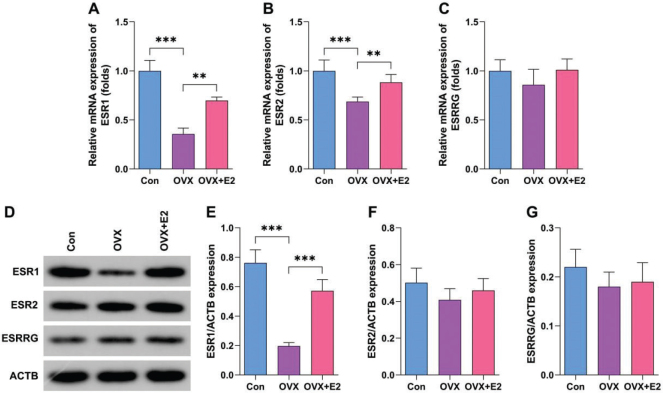
Estrogen predominantly upregulates estrogen receptor alpha (ESR1) in OVX mouse model. (A–D) mRNA levels in 12-week-old female C57BL/6J mice were analyzed by quantitative reverse transcriptase-polymerase chain reaction (qRT-PCR). (E–H) Protein expression in 12-week-old female C57BL/6J mice was analyzed using Western blot. **P < 0.01, ***P < 0.001.

Estrogen levels are regulated by its receptors, such as ESR1, ESR2, and estrogen-related receptor gamma (ESRRG). To confirm this, we determined the expression of these genes in OVX-induced OP models. We found that OVX significantly reduced the messenger RNA (mRNA) levels of ESR1 and ESR2 (Fig. 4A–B), particularly ESR1, which was reversed by estrogen administration. However, ESRRG expression showed no significant alteration (Fig. 4C). Furthermore, we found that estrogen administration significantly increased the protein expression of ESR1, but showed no significant effects on ESR2 and ESRRG expression (Fig. 4D–G). These results suggest that E2 predominantly upregulates ESR1 in OP.

### Estrogen promotes the interaction between ESR1 and RUNX2

**Fig. 5:**
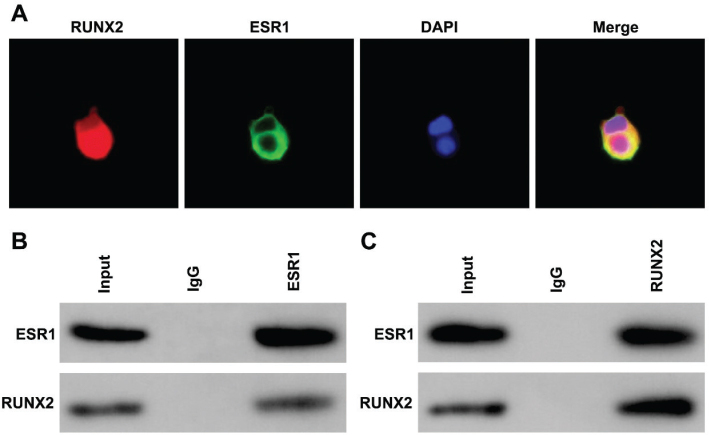
Estrogen promotes the interaction between estrogen receptor alpha (ESR1) and RUNX2. (A) The location of ESR1 and RUNX2 in bone marrow stromal cells (BMSCs) was analyzed by FISH assay. (B–C) The interaction between ESR1 and RUNX2 in BMSCs was analyzed by Co-IP assay.

Compared with the control group, ESR1 and RUNX2 were co-localized in the nuclei of BMSCs (Fig. 5A), suggesting that estrogen can promote the interaction between ESR1 and RUNX2. Furthermore, the co-immunoprecipitation (Co-IP) assay confirmed the interaction between ESR1 and RUNX2 (Fig. 5B–C).

### ESR1 promotes angiogenesis of BMSCs

**Fig. 6:**
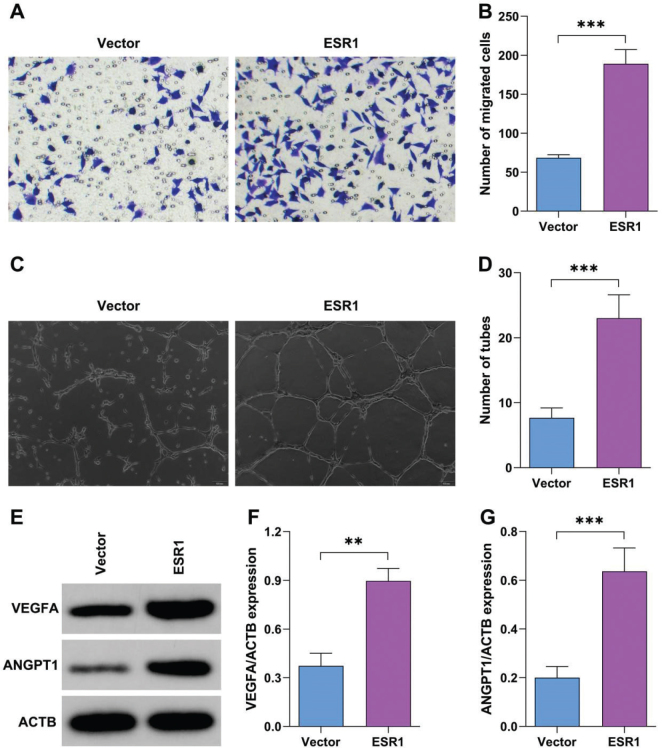
Estrogen receptor alpha (ESR1) promotes angiogenesis of bone marrow stromal cells (BMSCs). (A–B) The migration of BMSCs after transfection with ESR1 overexpression plasmids or empty vector was analyzed by Transwell assay. (C–D) Tube formation of BMSCs after transfection with ESR1 overexpression plasmids or empty vector was analyzed using the tube formation assay. (E–G) Protein expression after transfection with ESR1 overexpression plasmids or empty vector was analyzed using Western blot. **P < 0.01, ***P < 0.001.

To confirm the role of ESR1 in OP, BMSCs were transfected with ESR1 overexpression plasmids. ESR1 overexpression significantly increased the migrated cells of BMSCs (Fig. 6A–B). Moreover, ESR1 overexpression significantly increased the branches of BMSCs (Fig. 6C–D). ESR1 overexpression significantly increased the protein expression of VEGFA and ANGPT1 (Fig. 6E–G).

### Estrogen promotes osteogenesis of BMSCs

**Fig. 7:**
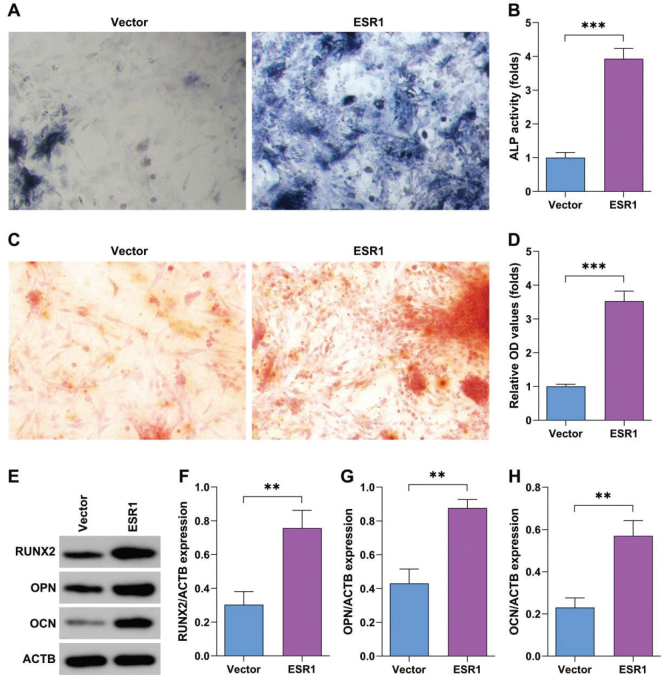
Estrogen promotes osteogenesis of bone marrow stromal cells (BMSCs). (A–B) The osteogenic ability of BMSCs after transfection with estrogen receptor alpha (ESR1) overexpression plasmids or empty vector was analyzed by alkaline phosphatase (ALP) staining. (C–D) Cellular mineralization of BMSCs after transfection with ESR1 overexpression plasmids or empty vector was analyzed using alizarin red S (ARS) staining. (E–H) Protein expression after transfection with ESR1 overexpression plasmids or empty vector was analyzed using Western blot. **P < 0.01, ***P < 0.001.

ESR1 overexpression significantly promoted the osteogenic differentiation of BMSCs compared with the control group (Fig. 7A–B). ESR1 overexpression significantly mediated cellular mineralization of BMSCs compared with the control group (Fig. 7C–D). ESR1 overexpression significantly increased the protein expression of RUNX2, OPN, OCN (Fig. 7E–H).

## Discussion

Low bone mineral density (BMD) and impaired bone microarchitecture are the main features of OP (24). Therefore, restoring osteogenic differentiation and bone microarchitecture may prove to be a promising strategy for OP. Accumulating strategy studies have evidenced that selective estrogen receptor modulators (SERMs) effectively alleviate the progression of OP (20, 25). Here, we demonstrated that estrogen improved BMD and bone microarchitecture. Furthermore, estrogen promoted the osteogenesis and angiogenesis of BMSCs. Mechanistically, estrogen upregulated ESR1, which interacted with the master regulator of osteogenesis. Therefore, estrogen can be a therapeutic strategy for OP.

Estrogen deficiency contributes to the pathogenesis of primary ovarian insufficiency and postmenopausal OP (26, 27), inducing imbalance between bone formation and resorption. For instance, estrogen deficiency contributes to inflammation response and osteoblastogenesis inhibition (28). Estrogen withdrawal-mediated iron accumulation promotes the ferroptosis of osteocytes in OP (29). Interestingly, it results in stimulating the release of estrogen proliferation and differentiation of osteoblast, inhibiting the progression of OP (30). Furthermore, estrogen mediates duodenal calcium absorption and ameliorates OP (31). In our study, estrogen treatment mediated bone formation and also promoted the osteogenesis and angiogenesis of BMSCs, manifested by the upregulation of osteogenesis-related marker (RUNX2, OPN, and OCN) and angiogenesis-related markers (VEGFA and ANGPT1) (32–34). These findings are consistent with previous studies that stated the fact that estrogen effectively promotes osteoblastic bone formation and angiogenesis (23, 35–37). These findings suggested that estrogen plays a protective role in OP.

Estrogens, via ESR1, are important in the development and maintenance of bone mineral density (38). Indeed, ESR1 collectively participates in regulating bone mass (39). For instance, Suthon’s study reports that E2-mediated activation of ESR1 signaling protects against bone loss in OP (40). ESR1-expressing osteoblast increases cortical bone thickness as well as trabecular bone mass (41). However, ESR1 depletion contributes to a decrease of cortical bone mass (42). Here, we demonstrated that ESR1 was downregulated in OP. Furthermore, estrogen predominantly upregulated ESR1. ESR1 overexpression mediated osteogenesis and angiogenesis of BMSCs.

A previous study evidences that nuclear estrogen receptor mediates the increase of cortical bone mass (43). Here, we demonstrated that ESR1 and RUNX2 were co-localized in nuclear of BMSCs, which was enhanced by E2. RUNX2, as the master regulator of osteogenesis, is frequently downregulated in OP (44). Interestingly, restoring RUNX2 expression alleviates the pathogenesis of OP. For instance, Yam-derived exosome-like nanovesicles inhibit OP by activating BMP2/RUNX2 signaling (45). NIBAN2-mediated nuclear localization of RUNX2 antagonizes the progression (46). Here, we demonstrated that E2 enhanced nuclear localization of ESR1 and RUNX2. Jeong et al. (47). provided evidence that ESR1 interacts with RUNX2 to maintain osteoblast lineage cell function as well as bone formation. Therefore, estrogen may protect against OP by enhancing the interaction between ESR1 and RUNX2.

Osteogenesis is indispensable for new bone regeneration, but these processes frequently decline in ageing (2, 48). Recently, the osteogenesis–angiogenesis coupling theory has been receiving increasing attention (49–51). Besides osteogenesis, bone vasculature growth maintains perivascular osteoprogenitors, and couples angiogenesis to osteogenesis. In the treatment of OP, osteogenesis–angiogenesis coupling of BMSCs enhances osteoporotic bone repair (52). Activation of RUNX2 signaling drives osteogenesis–angiogenesis coupling and osteoporotic bone regeneration (53). Therefore, estrogen promotes osteogenesis–angiogenesis coupling in OP by activating ESR1/RUNX2 signaling.

In conclusion, estrogen exerts protective effects on OP. Estrogen promotes the angiogenesis and osteogenesis of BMSCs by enhancing the interaction between ESR1 and RUNX2. Therefore, estrogen may prove to be a promising strategy for OP.

## Declarations

### Availability of data and material

The data sets used and analyzed in the current study are available on reasonable request from the corresponding authors.
